# The Book World of Medicine and Science

**Published:** 1906-08-25

**Authors:** 


### The Book World of Medicine and Science.

A Handbook of Diseases of the Nose and Pharynx. By James B. Ball, M.D., Physician to the Department for Diseases of the Throat, Nose, and Ear at the West London Hospital, etc. Fifth edition. Small octavo, with seventy-eight illustrations. (London : Baillière, Tindall, and Cox, 8 Henrietta Street, Covent Garden. Price 7s. 6d. net.)

The fifth edition of this work, as might be expected from the high reputation of its author, is an excellent short manual of the department of medical knowledge of which it treats. It is written in an elegant and lucid style, and is very well arranged. Another good point is that there are no redundancies; the book is thus kept small, and is presented to the reader within the compass of less than 400 pages. The time is past when diseases of the nose and pharynx were considered to be of trifling importance, and it is now recognised that the young practitioner is not fully equipped without some knowledge of them. The work before us is admirably adapted to fulfil the aim of its author in supplying this need. Incidentally, too, it does more than this, for the various subjects are so treated as to make the work full of suggestiveness to the thoughtful reader, and a number of interesting questions, of necessity left unanswered in its pages, offer themselves during its perusal. What, for instance, is the relationship between the local disorder of the nose or pharynx and the general disturbance of health accompanying it? In certain cases it is clearly demonstrated that a profound effect upon the general well-being may be produced by an abnormal condition of a purely local nature, such as nasal obstruction, nasal suppuration, or the presence of adenoids in the naso-pharynx. To one unacquainted with this fact the ultimate cause of repeated colds, of bronchial attacks, of dyspepsia, of asthma, or of such a symptom as headache, may be entirely overlooked in a certain proportion of cases. But besides these instances, in which the sequence is obvious, there are others where the relationship is not at all so clear. In fœtid atrophic rhinitis or ozæna, for example, the local symptoms are sometimes accompanied by a disturbance of health, or, as the author tells us, by so-called strumous signs. According to some writers, "genuine" ozæna is due to an obscure constitutional cause, while other cases of "pseudo-ozæna," with precisely similar local symptoms, are produced by local causes. Upon this question the author, while not discarding the hypothesis of a "genuine" ozæna, of which he states very truly that the diagnosis must be cautiously made, says : "It is a question whether the anæmia and the malnutrition which are so often present in these patients are not a result rather than a cause of the disease." He alludes also to the important work of Grünwald, who endeavours to prove that ozæna is always due to a focus of suppuration either in the nose itself, in the naso-pharynx (adenoids, for example), or else in the accessory sinuses. This writer is commonly represented as saying that the focus is always in one or more of the accessory cavities, but besides being a much less tenable position this is a mistake. In regard to the treatment of ozæna Dr. Ball very judiciously insists upon the paramount importance of local measures. Efficient cleansing with simple solvent lotions is essential; disinfecting lotions are really unnecessary. It might be added that irrigation of the nose with strong carbolic or other powerful antiseptic lotions, as we have known to be done, is most injurious. Only a very short mention is made of the treatment by paraffin injections into the turbinal bodies, advocated by French and Belgian rhinologists, who appear to have had very good results. The weakest page of rhinology is certainly that relating to etiology, probably because the discovery of causes is so very difficult. What, for instance, are the causes of adenoid vegetations? Although a number of interesting facts having a bearing upon etiology are discussed, no explanation is given either here or elsewhere of the causation of this very common condition. This being so, we demur slightly to the statement that most cases of so-called recurrence are undoubtedly due to imperfect operation. It may be so in some cases, but if the liability to the complaint is hereditary we should expect it to recur, for the whole of the lymphoid tissue in the naso-pharynx is not removed even by the most perfect operation. In any case, the unknown causes may continue to act undisturbed even after the removal of their effects. The question as to cause may also be asked with regard to hypertrophy of the tonsils, attributed by the author in the majority of cases to a special predisposition to hypertrophy of adenoid structures; and to hypertrophy of the lingual tonsil, upon the etiology of which he frankly states that nothing dennite is known. The truth is that the why and the wherefore of these lymphoid hypertrophies is yet but very imperfectly understood. Whether they are due to some obscure constitutional conditions, or to some local causes, which are yet unrecognised, and how those causes operate are questions which still remain to be unravelled. In the meantime, it is perhaps safer for the student of rhinology to assume the existence of local causes for purely local affections, and to search diligently for these, rather than to treat some hypothetical general condition for the existence of which no shadow of proof exists. Want of space forbids us to digress further in the regions of speculation. We may, however, mention the chapter upon septal deformities, in which Moure's operation is not even named, so completely has resection taken its place for all cases in the author's estimation; also the excellent account of sinus disease, and, amongst diseases of the pharynx, chronic pharyngitis which, deservedly, has a chapter to itself. To all the good points in the book it is impossible to allude : it needs reading with care, as frequently some important observation is contained in a single sentence. There is one feature which should make the work of special value to the practitioner, namely the care and attention devoted throughout to the subject of treatment. Besides a section upon general treatment in the first part, the best modes of dealing with all the various affections in turn are fully described, with many suggestions from the author's personal experience, and at the end of all is a short but well-selected collection of formulæ.

Surgical Suggestions : Practical Brevities in Diagnosis and Treatment. By Walter M. Brickner, M.D. and Eli Moschcowitz, M.D. (New York : Surgery Publishing Company, 92 William Street, 1906.)

Some of the "Surgical Suggestions" are scintillations, but most of them are mere platitudes. Such isolated portions of surgical knowledge are of but little use, unless the mind has been well stored as a result of full reading, when they may serve to start fresh trains of thought while ruminating on the particular topic so tersely epitomised. The fault that we find is that these literary condensations are far away too brief and too few—two concise comments only on the whole field of cranial surgery! and the huge subject of Rhinological Science is summarily despatched with only four scraps. These surgical tit-bits may serve to interest and instruct many of our readers who still cherish the epigrammatic. The litle book is well arranged, printed, and finished, and we can recommend it for pastime purposes.

